# Research on Visual Perception of Speed Bumps for Intelligent Connected Vehicles Based on Lightweight FPNet

**DOI:** 10.3390/s24072130

**Published:** 2024-03-27

**Authors:** Ruochen Wang, Xiaoguo Luo, Qing Ye, Yu Jiang, Wei Liu

**Affiliations:** 1 School of Automotive and Traffic Engineering, Jiangsu University, Zhenjiang 212013, China; xiaoguoluo0206@163.com (X.L.); jyu626@126.com (Y.J.); ujsliuwei@163.com (W.L.); 2 Automotive Engineering Research Institute, Jiangsu University, Zhenjiang 212013, China; yeqing0610@163.com

**Keywords:** intelligent connected vehicles, autonomous driving, visual perception, speed bumps, YOLOv5, deep learning

## Abstract

In the field of intelligent connected vehicles, the precise and real-time identification of speed bumps is critically important for the safety of autonomous driving. To address the issue that existing visual perception algorithms struggle to simultaneously maintain identification accuracy and real-time performance amidst image distortion and complex environmental conditions, this study proposes an enhanced lightweight neural network framework, YOLOv5-FPNet. This framework strengthens perception capabilities in two key phases: feature extraction and loss constraint. Firstly, FPNet, based on FasterNet and Dynamic Snake Convolution, is developed to adaptively extract structural features of distorted speed bumps with accuracy. Subsequently, the C3-SFC module is proposed to augment the adaptability of the neck and head components to distorted features. Furthermore, the SimAM attention mechanism is embedded within the backbone to enhance the ability of key feature extraction. Finally, an adaptive loss function, Inner–WiseIoU, based on a dynamic non-monotonic focusing mechanism, is designed to improve the generalization and fitting ability of bounding boxes. Experimental evaluations on a custom speed bumps dataset demonstrate the superior performance of FPNet, with significant improvements in key metrics such as the mAP, mAP50_95, and FPS by 38.76%, 143.15%, and 51.23%, respectively, compared to conventional lightweight neural networks. Ablation studies confirm the effectiveness of the proposed improvements. This research provides a fast and accurate speed bump detection solution for autonomous vehicles, offering theoretical insights for obstacle recognition in intelligent vehicle systems.

## 1. Introduction

In modern transportation systems, speed bumps are used for traffic management to slow down vehicles and improve pedestrian safety [1]. However, the target identification algorithms of autonomous driving systems often ignore speed bumps when identified, resulting in the vehicle driving over speed bumps at high speeds. This presents a risk of rollover and collision, endangering the safety of the vehicle occupants and pedestrians [2]. Additionally, this impacts the comfort of the vehicle occupants [3]. Therefore, it is crucial for autonomous vehicles to identify speed bumps accurately and promptly.

Conventional speed bump detection typically relies on mathematical models of vehicle dynamics or complex image processing algorithms and costly sensors [4,5]. These methods either lack predictability by only detecting the current road surface or require a large number of computational resources, making it difficult to meet the real-time performance requirements of autonomous driving systems. In recent years, deep learning techniques have rapidly developed and provided new solutions for speed bump identification. Specifically, methods based on convolutional neural networks (CNNs) have demonstrated good detection performance in a variety of traffic scenarios. Deep learning-based target identification algorithms can be further divided into two-stage methods and one-stage methods [6,7].

Representative works of two-stage object detection algorithms include the R-CNN series [8,9,10,11,12], which are fundamentally characterized by first extracting candidate regions from images, followed by the classification of these regions. Thus, the exceptional performance in accuracy for object detection tasks is exhibited with this category of algorithms. Two-stage methods achieve high accuracy in target identification tasks, reaching 85.6% on the VOC2007 dataset, but due to the need to perform the inference twice, they fall short in terms of processing speed, reaching only 15 frames per second (FPS). Contrastingly, single-stage object detection algorithms, such as You Only Look Once (YOLO) [13] and Single Shot Multibox Detector (SSD) [14], are characterized by a more direct strategy. Target boxes and their corresponding categories are predicted at multiple locations in the image through a single inference process by these algorithms. This method optimizes computational efficiency and dramatically improves processing speed, achieving an FPS of up to 100. However, it falls slightly short in detection accuracy compared to the two-stage methods, with an accuracy of only 75.1% on the VOC2007 dataset.

However, deep learning-based models often have a large number of parameters and high computational complexity, which can be a significant constraint for autonomous driving systems with limited computational capability. Existing pavement identification algorithms commonly utilize semantic segmentation in target identification, accurately identifying the boundaries between obstacles and drivable areas on the road. However, the algorithms’ FPS is limited to 40, resulting in poor real-time performance. To address the challenge of balancing real-time challenges and accuracy in target identification algorithms, the use of lightweight models in autonomous vehicle driving systems has emerged as a crucial research area in academia. To address the aforementioned issues, scholars have proposed solutions, such as PVANet [15] and SSDRNet [16]. These lightweight models aim to balance computational resources with speed and accuracy in identification tasks. Although studies on lightweight modeling applications address limited computational resources, maintaining a balance between detection speed and accuracy remains challenging. Therefore, designing a new high-precision lightweight neural network model that can be deployed on edge devices holds significant value, both in research and in practicality.

Due to its computational efficiency and ease of use, the YOLO framework has gained widespread attention in academia and industry as a modeling tool for target identification. The framework is composed of three parts: the backbone, neck, and head, each containing different modules. An attention mechanism is employed to mimic the way humans focus on important features while ignoring distracting information. The loss function is a method in deep learning that is used to evaluate the differences between the predictions of a model and the actual results. Through the loss function, the parameters of the model can be constantly adjusted, so that the model’s predictions are closer to the real label.

To address the deficiencies of neural network models in detecting speed bumps, this study proposes an enhanced lightweight object detection network model, YOLOv5–Faster&Preciser Net (YOLOv5-FPNet). The development of the model is based on the following: Firstly, the architecture integrates FasterNet with Dynamic Snake Convolution, effectively reducing the parameter count and floating-point operations of the backbone network while significantly enhancing detection accuracy. Subsequently, an innovative modification to the C3 module in the head is proposed as the C3–SerpentFlowConv (C3-SFC) module. The aim is to enhance the ability of the neck and head components to adapt to distorted features. Furthermore, a parameter-free attention mechanism SimAM is embedded in the backbone network and the large target detection head, with the aim of enhancing the feature extraction capability of the backbone and the detection head. Finally, the fitting and generalization capabilities of the model for predicted targets are bolstered through the introduction of Inner–WIoU. Through ablation and comparison experiments, FPNet is verified to have better performance on speed bump detection tasks, demonstrating its significant advantages in the field of target detection.

In summary, the principal contributions of this study are as follows:

i. A deformable lightweight neural network model, FPNet, is introduced, which is capable of more effectively addressing detection failures caused by image distortions.
ii. The C3-SFC module is proposed to enhance the feature expression capability of the C3 module. Furthermore, the SimAM attention mechanism is embedded within both the backbone network and the large-object detection head to improve the feature extraction capability of the model and suppress the interference caused by background noise.
iii. Inner–WiseIoU is proposed to optimize the fitting and generalization capabilities of the anchor box.

## 2. Related Works

Given that the FPNet proposed in this study is a lightweight neural network model tailored for real-time speed bump detection, this section provides a review of speed bump detection methodologies and existing lightweight neural network models.

### 2.1. Speed Bump Detection Methods

Currently, there are numerous methods for speed bump identification based on mathematical models of vehicle dynamics, and there are also methods based on deep learning. Methods that utilize mathematical models of vehicle dynamics achieve speed bump identification by analyzing the dynamic response of vehicles under various road conditions, including acceleration, vibration, and other relevant characteristics. These methods include an artificial intelligence-based pavement identification method proposed by Qin et al. [17,18]; a logic model for detecting speed bumps using genetic algorithms proposed by Celaya-Padilla et al. [19]; a method for estimating pavement elevation using fuzzy logic proposed by Qi et al. [20]; and a method for applying a short-time Fourier transform to estimate pavement elevation proposed by Domínguez et al. [21]. It should be noted that the methods based on a mathematical model of vehicle dynamics mentioned above can achieve an accuracy of over 97%. However, it is important to keep in mind that these methods are only able to conduct vehicle identification in a quasi-real-time manner. This means that the vehicle can only be accurately identified after it has passed over the speed bumps, and there is a time delay. As speed bumps are an unconventional road condition, quasi-real-time identification cannot satisfy the needs of autonomous vehicles for advanced sensing, advanced decision-making, and timely execution.

Compared to the methods mentioned above, deep learning-based speed bump identification methods can learn the feature representation of speed bumps automatically, which means that deep learning-based speed bump identification models have better generalization ability. There are two main categories for identifying speed bumps using CNNs: semantic segmentation-based and anchor box-based. Semantic segmentation-based methods include SegNet, first proposed by Vijay et al. [22] and applied to speed bump identification work by Arunpriyan et al. [23]. Varona et al. [24] proposed a method based on CNNs, Long Short-Term Memory Networks (LSTMs), and Reservoir Computing Models. There has also been a speed bump identification algorithm for Microsoft Kinect sensors proposed by Lion et al. [25] as well as a Gaussian filtering-based speed bump identification proposed by Devapriya et al. [26]. Anchor box-based methods include a speed bump detection method combining LiDAR and a camera proposed by Yun et al. [27]. A GPU and ZED stereo camera-based speed bump identification method was proposed by Varma et al. [28], and. Dewangan et al. [29] proposed a model for speed bump detection based on deep learning and computer vision. All of these methods can achieve approximately 85% accuracy.

It should be noted that while the semantic segmentation-based approach achieves a maximum accuracy of 89%, the computationally intensive nature of pixel-level segmentation restricts speed processing to a maximum of only 30 FPS. Additionally, the anchor box-based speed bump identification algorithm performs slightly better in terms of FPS, reaching 30–60 FPS but experiences issues due to poor anchor box convergence and limited detection distance.

In advanced autonomous driving systems, a processing speed of 100 FPS is considered standard to effectively capture dynamic changes [30]. This ensures that an autonomous driving vehicle can make decisions and react at the millisecond level, significantly improving safety. However, CNN-based methods do not meet the stringent requirements for millisecond-level decision making in autonomous driving systems due to their low processing speeds.

### 2.2. Lightweight Neural Network Model

In 2016, Iandola et al. [31] proposed SqueezeNet, which introduces the Fire module to perform Squeeze and Expand operations. The Squeeze operation reduces the number of channels using a 1 × 1 convolution kernel. The Expand operation enhances the model’s expressive power by combining a 1 × 1 convolution kernel with a 3 × 3 convolution kernel to increase the number of channels.

In 2017, Google proposed the MobileNet [32] model, which for the first time proposed the concept of a lightweight neural network model. The model computation is significantly reduced by using depth-separable convolution, which is a decomposition of the traditional convolution operation into depth convolution and point-by-point convolution. After MobileNet, Google released MobileNetV2 [33] in 2018, which introduced a linearized neck layer structure and optimized the activation function, an improvement that made the model more efficient in computationally resource-constrained environments. MobileNetV3 [34], proposed in 2019, further optimized the series by adding SE modules to the backbone and optimizing the activation function and output structure, thus significantly enhancing performance.

In 2017, Chollet [35] proposed Xception, which utilized deep separable convolution technology. Xception has a modular design that enhances model flexibility and introduces residual connections to address the issue of gradient vanishing during model training. This improves the stability and efficiency of the training process.

In 2018, Zhang et al., from MEGVII Technology, proposed ShuffleNet [36], which is a model that effectively achieved more efficient feature delivery, reduced computational complexity, and improved the computational efficiency of the model through the introduction of point-by-point grouped convolution and channel shuffling mechanisms. After ShuffleNet, Ma et al., also from MEGVII Technology, introduced ShuffleNetV2 [37]. This model proposed ShuffleUnit, which combined residual connectivity and channel-by-channel convolution to reduce the fragmentation of the model and achieve a significant improvement in performance.

In 2020, GhostNet was proposed by Han et al. [38] from the University of Sydney. This model utilizes simple linear operations on feature maps to generate additional similar feature maps, effectively reducing the parameter count. GhostNet innovates by replacing traditional convolutions with the Ghost Module, using standard 1 × 1 convolutions for channel count compression of the input image, followed by depthwise convolutions for additional feature map extraction and, finally, concatenating various feature maps to form a new output.

Notwithstanding the outstanding contribution of the above models to lightweighting neural networks, which reduces the number of parameters and computational complexity of the models, they also face some challenges such as the degradation of recognition accuracy, limited feature expressiveness, and difficulties in the training process.

## 3. Methodology

**Figure 1:**
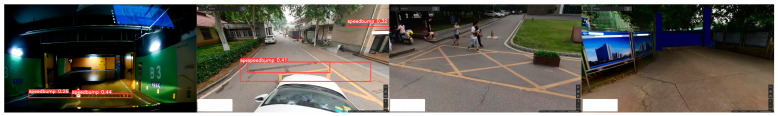
Detection results of YOLOv5.

The YOLO algorithm, proposed by Joseph Redmon et al. [13] in 2016, has been optimized over the years to enhance the detection accuracy and computational efficiency of the algorithm. Despite the YOLO series [39,40,41] has maintained a leading position in the real-time target detection field and demonstrated excellent performance in several application scenarios, there still exist limitations. Firstly, YOLOv5 still has much space for optimization in the balance between real-time and detection accuracy. Secondly, YOLOv5 is not sufficiently robust when dealing with targets that have significant aspect ratio variations, image distortions, and occluded targets; thus, there is the problem of missed detection. Finally, the model has limited adaptability to complex scenarios and is susceptible to background noise and complex lighting conditions, leading to unstable detection results. The YOLOv5 detection results are shown in Figure 1.

To address the above issues, the following strategies are proposed in this study as a countermeasure.

**Figure 2:**
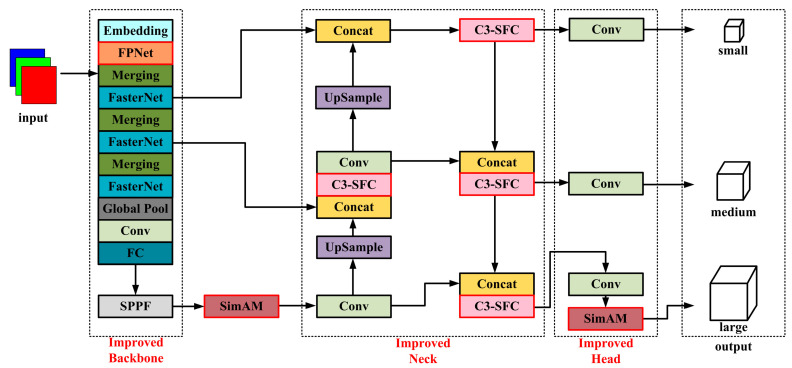
Schematic diagram of YOLOv5-FPNet network structure. Different colors represent different modules.

Firstly, to address the problems of YOLOv5 in terms of balancing real-time and detection accuracy as well as the missing detection of targets with significant aspect ratio variations and image distortions, FPNet is proposed using FasterNet [42] integrated with Dynamic Serpentine Convolution (DSConv) and replaced with the original backbone network, which ensures the detection accuracy of the model while reducing the number of model parameters and the amount of floating-point operations, as well as using adaptive variation to accurately extract structural features of targets with significant aspect ratio variations or image distortions. Subsequently, to address the issues of insufficiently robust processing when handling occluded targets and limited adaptability for complex scenarios, the SimAM [43] attention mechanism is embedded in the backbone network and the large target detection head, and the C3-SFC module is proposed to enhance the feature extraction capability. Finally, an adaptive loss function based on a dynamic non-monotonic focusing mechanism, *Inner–WiseIoU*, is proposed to enhance the fitting and generalization ability of the anchor box. The proposed YOLOv5-FPNet is shown in Figure 2.

### 3.1. Design of FPNet

Chen et al. [42] highlighted a major problem with lightweight neural networks: While numerous lightweight models have successfully reduced the number of parameters, most of them still rely on frequent memory accesses, which lengthens the execution time of the model. Comparatively, models that have reduced the frequency of memory accesses, such as MicroNet [44], exhibit the problem of relying on inefficient fragmented computation, which contradicts the intended purpose of lightweight designs.

**Figure 3:**
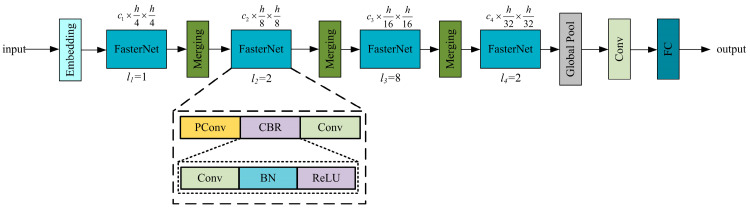
Schematic diagram of FasterNet structure.

To address this problem, Chen et al. [42] proposed FasterNet, which avoids unnecessary redundant operations, achieves a better balance between accuracy and real-time performance, and demonstrates great potential in edge device applications. A schematic diagram of the FasterNet structure is shown in Figure 3.

**Figure 4:**
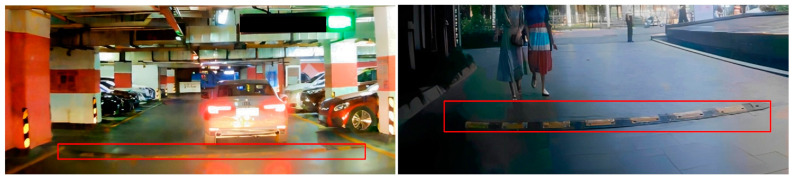
Example of image distortion. The speed bumps in the red box show the image distortion clearly.

However, when FasterNet was applied as a backbone for the experiment, the problem of missed detection of significant targets was observed. Missed targets are often caused by image distortion, resulting from the lens effect of the camera. This means that straight objects at the edge of the image may appear curved. Image distortion is a common occurrence during image acquisition, as shown in Figure 4.

To address the above issues, this study adds deformable convolution kernels [45,46] based on FasterNet. The Dynamic Snake Convolution (DSConv) proposed by Qi et al. [47] is used to improve the model’s ability to handle object shapes and details.

DSConv has demonstrated excellent performance in recognizing tubular objects because the flexibility of its convolutional kernel allows it to “stravaig” around the target object, efficiently adapting and accurately capturing the features of the tubular structure. Thus, the model’s performance, in terms of complex scenes and deformed objects, is improved.

**Figure 5:**
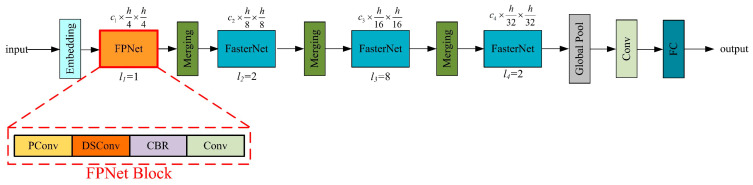
Schematic diagram of FPNet network structure.

The structure of the proposed FPNet network after the addition of DSConv to FasterNet is shown in Figure 5.

**Figure 6:**
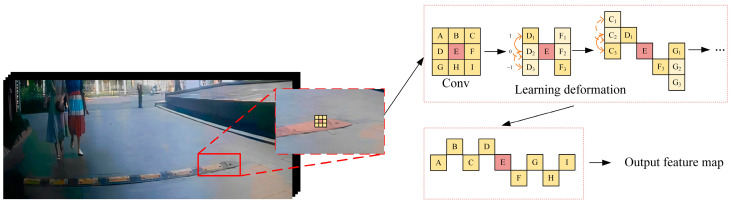
Deformation mechanism of DSConv.

The deformation mechanism of DSConv is shown in Figure 6.

The deformation rule of DSConv along the *x*-axis is as follows:

(1) $$K_{i\pm c}=\left\{ \begin{array}{l} (x_{i+c},y_{i+c})=(x_{i}+c,y_{i}+\sum_{i}^{i+c}\Delta y)\\ (x_{i-c},y_{i-c})=(x_{i}-c,y_{i}-\sum_{i-c}^{i}\Delta y) \end{array}\right.$$

The deformation rule along the *y*-axis is as follows:

(2) $$K_{i\pm c}=\left\{ \begin{array}{l} (x_{j+c},y_{j+c})=(x_{j}+c,y_{j}+\sum_{j}^{j+c}\Delta x,y_{j}+c)\\ (x_{j-c},y_{j-c})=(x_{j}-c,y_{j}-\sum_{j-c}^{j}\Delta x,y_{j}-c) \end{array}\right.$$

The offset Δ is typically a fraction, and the implementation of bilinear interpolation is as follows:

(3) $$K=\sum_{\overset{\cdot }{K}}B(K^{\prime },K)\cdot K^{\prime }$$

where *K* denotes the position of the fraction in Equations (1) and (2), *K’* denotes all the enumerated integral spatial positions, and *B* denotes the bilinear interpolation kernel. The bilinear interpolation kernel is divided into two one-dimensional kernels:

(4) $$B(K,K^{\prime })=b(K_{x},{K^{\prime }}_{x})\cdot b(K_{y},{K^{\prime }}_{y})$$

**Figure 7:**
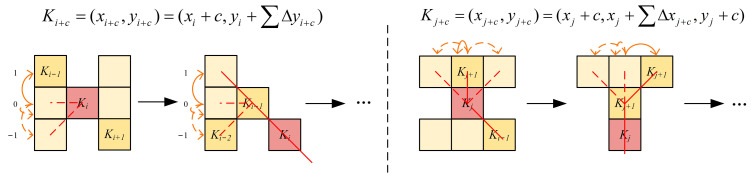
Schematic diagram of *x*- and *y*-axis coordinate deformation.

The deformation of the *x*-axis and *y*-axis coordinates is illustrated in a schematic diagram shown in Figure 7.

**Figure 8:**
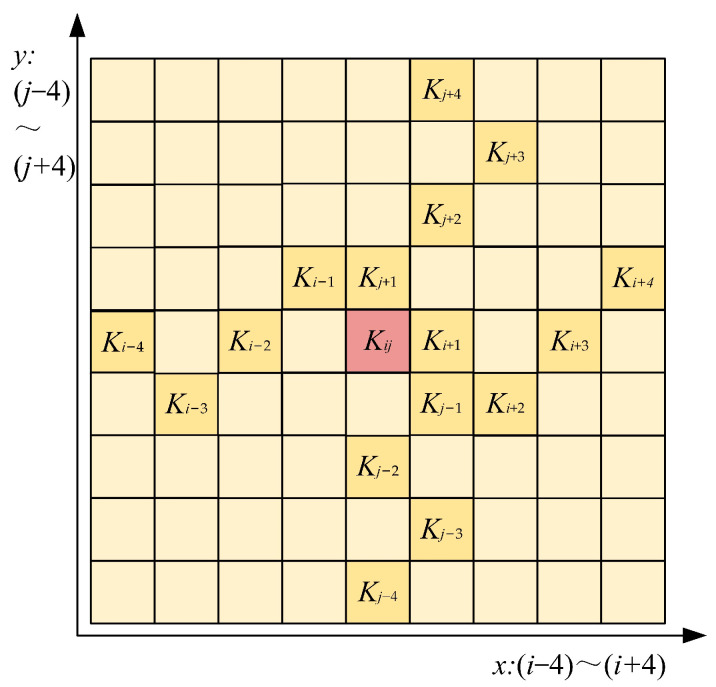
Receptive field of DSConv.

Under the assumption that the range of values on the *x*-axis is [*i* − 4, *i* + 4] and the range of values on the *y*-axis is [*j* − 4, *j* + 4], the sensibility field of DSConv in this range of values is shown in Figure 8.

Figure 8 shows that after coordinate deformation on the *x*- and *y*-axis, DSConv is able to obtain a receptive field that can cover an area of 9 × 9 for better perception of key features.

In order to fully evaluate the performance of DSConv, this thesis implemented a convolutional kernel performance comparison experiment. The experiment involved constructing a simple neural network model to which DSConv was applied. In order to establish the effectiveness and superiority of DSConv, the experiment specifically compared it to the currently dominant convolutional kernel. The detailed results of the experiment are collated and presented in Table 1, and these data provide a quantitative basis for evaluating the performance of DSConv across different aspects.

According to the data given in Table 1, DSConv has smaller parameters and almost the same FPS compared to the traditional Conv2d convolution kernel. Thus, DSConv achieves a good balance between accuracy and computation.

In summary, FPNet successfully solves the target loss problem due to image radial distortion and significantly improves the overall performance of the backbone in speed bump detection by introducing the deformation mechanism of DSConv into FasterNet.

### 3.2. Design of C3-SFC Module

In the neck section of YOLOv5, the CSP_3 (C3) module is used to increase the depth and receptive field of the neural network model. This module’s design greatly enhances the network’s ability to extract features, allowing it to process and recognize complex image content more efficiently, resulting in improved accuracy and robustness of detection. However, the operation of the C3 module generates a large number of parameters, which can significantly drain memory resources and lead to problems such as vanishing gradients and information bottlenecks. These issues can limit the overall performance of the neural network model.

**Figure 9:**
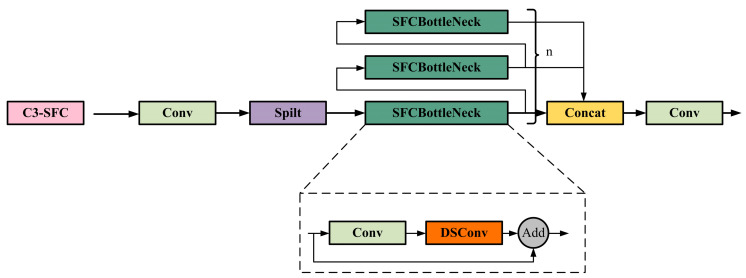
Schematic diagram of C3-SFC module.

To tackle these challenges, this study has improved the C3 module by drawing on the Efficient Layer Aggregation Network (ELAN) concept proposed by Wang et al. [50]. The study introduced the DSConv operator to the BottleNeck section of the C3 module, resulting in the creation of the SFCBottleNeck. Based on this enhancement, the study further introduces the C3-SFC module, which aims to improve the model’s efficiency and performance through structural optimization. Figure 9 shows the detailed architecture of the enhanced C3-SFC module.

Compared to the traditional Conv2D convolution operator, DSConv offers significant advantages in terms of parameter and floating-point operation reduction. Table 1 shows a 62.59% reduction in parameters and an 87.01% reduction in floating-point operations. Based on these data, it can be concluded that SFCBottleNeck outperforms the traditional BottleNeck structure in terms of parameters and operations. These improvements enable SFCBottleNeck to effectively increase the computational speed and accuracy of the original C3 module.

### 3.3. Attention Mechanism

This study introduces the Simulation Attention Module (SimAM) proposed by Yang et al. [43] to address the problem of lighting conditions and background noise affecting model detection accuracy in road target detection tasks. The purpose of SimAM is to enhance the ability of neural network models to concentrate on crucial areas in an image. This attention mechanism enables the network to more accurately identify and process important features of the target, thereby improving the accuracy and reliability of detection under complex environmental conditions.

**Figure 10:**
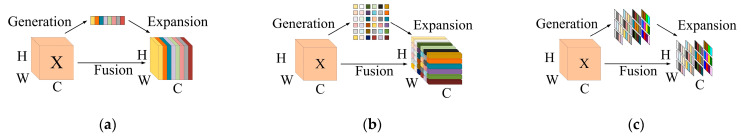
SimAM schematic diagram: (**a**) one-dimensional channel attention, (**b**) two-dimensional spatial attention, and (**c**) three-dimensional spatial weighted attention.

SimAM achieves higher efficiency and smaller attention weights by utilizing the linear separability between neurons, without introducing additional parameters. The mechanism identifies key neurons and prioritizes them for attention, resulting in efficient feature map extraction. The principle of operating SimAM is illustrated in Figure 10.

In this study, SimAM is embedded in the backbone network and the large target detection head in YOLOv5-FPNet to enhance target detection performance.

The theoretical basis of SimAM is derived from neuroscience and uses an energy function to define the linear separability between a neuron *t* and every other neuron in the same channel except *t*. This approach successfully differentiates the relative importance of neurons and implements an effective attention mechanism. Equation (5) determines the energy function for each neuron:

(5) $$e_{t}(w_{t},b_{t},y,x_{i})={\left(y_{t}-\hat{t}\right)}^{2}+\frac{1}{M-1}\sum_{i=1}^{M-1}{\left(y_{0}-{\hat{x}}_{i}\right)}^{2}$$

where *t* represents the target neuron in a single channel of the input feature, and *x_i_* represents other neurons in the same channel. $\hat{t}$ and ${\hat{x}}_{i}$ denote the results after *t* and *x_i_* undergo linear transformation, where *i* indicates the spatial dimension index. The weight and bias of the linear transformation for the target neuron *t* are represented using $\hat{t}$ and ${\hat{x}}_{i}$, respectively. *λ* is identified as a hyperparameter, and *M* indicates the total number of neurons in a single channel.

$\hat{t}$ and ${\hat{x}}_{i}$ can be obtained from the following equation:

(6) $$\hat{t}=w_{t}t+b_{t}$$

(7) $${\hat{x}}_{i}=w_{t}x_{i}+b_{t}$$

By minimizing Equation (5), the equation is made equivalent to the linear separability between the target neuron *t* and other neurons in the same channel. To simplify Equation (5), *y_t_* and *y*_0_ are marked using binary notation, and a regularization matrix is added to Equation (5), resulting in the final energy function, as shown in Equation (8):

(8) $$e_{t}(w_{t},b_{t},y,x_{i})=\frac{1}{M-1}\sum_{i=1}^{M-1}[-1-(w_{t}x_{i}+b_{t})]+{\left[1-(w_{t}t+b_{t})\right]}^{2}+\lambda {w_{t}}^{2}$$

The linear transformation weight *w_t_* and the linear transformation bias for neuron *t* are determined using Equations (6) and (7), respectively.

(9) $$w_{t}=-\frac{2(t-\mu_{t})}{{\left(t-\mu_{t}\right)}^{2}+2{\sigma_{t}}^{2}+2\lambda }$$

(10) $$b=-\frac{1}{2}\cdot (t+\mu_{t})w_{t}$$

where *μ_t_* represents the mean of all neurons excluding neuron *t*, and *σ_t_*^2^ represents the variance of all neurons excluding neuron *t*.

The mean *μ_t_* of all neurons excluding neuron *t* and the variance *σ_t_*^2^ of all neurons excluding neuron *t* can be determined using Equations (8) and (9), respectively.

(11) $$\mu_{t}=\frac{1}{M-1}\sum_{i=1}^{M-1}x_{i}$$

(12) $${\sigma_{t}}^{2}=\frac{1}{M-1}\sum_{i=1}^{M-1}{\left(x_{i}-\mu_{t}\right)}^{2}$$

Assuming all pixels in a single channel follow the same distribution, the mean and variance of all neurons are calculated based on this assumption. These values are then reused across all neurons in that channel, significantly reducing the floating-point operation volume. The minimum energy e_t_^*^ is determined using the following formula:

(13) $${e_{t}}^{*}=\frac{4({\hat{\sigma }}^{2}+\lambda )}{{\left(t-\hat{\mu }\right)}^{2}+2{\hat{\sigma }}^{2}+2\lambda }$$

where ${\hat{\sigma }}^{2}$ represents the variance including neuron *t*, and $\hat{\mu }$ represents the mean including neuron *t*; these values can be determined using the subsequent equations:

(14) $$\hat{\mu }=\frac{1}{M}\sum_{i=1}^{M}x_{i}$$

(15) $${\hat{\sigma }}^{2}=\frac{1}{M}\sum_{i=1}^{M}{\left(x_{i}-\hat{\mu }\right)}^{2}$$

Utilizing Equation (13), it is understood that the value of the energy function is inversely correlated with the linear separability between neuron *t* and other neurons. This indicates that linear separability increases as the energy function value decreases. The design of the SimAM attention mechanism is guided by the energy function e_t_^*^, thus effectively avoiding unnecessary heuristics and adjustments. By computing for individual neurons and integrating the concept of linear separability into the entire model, a significant enhancement in the model’s learning capability is achieved.

### 3.4. Inner–WiseIoU

Intersection over Union (*IoU*) is used to measure the similarity between predicted bounding boxes and actual annotations, with a primary focus on the amount of region overlap between predictions and actual conditions.

Most existing works, such as *GIoU* [51], *CIoU* [52], *DIoU* [52], *EIoU* [53], and *SIoU* [54], assume that instances in training data are of high quality and focus on enhancing the fitting ability of Bounding Box Regression (BBR) loss. However, indiscriminately strengthening BBR on low-quality instances can lead to a decline in localization performance, as some researchers have discovered. In 2023, Tong et al. [55] built upon the static focusing mechanism (FM) proposed in *Focal-EIoU* [53] and introduced a loss function with a dynamic non-monotonic focusing mechanism called *Wise–IoU* (*WIoU*).

Since training data often include low-quality samples, this can result in a higher penalty for such samples, which can have a negative impact on the model’s ability to generalize. A well-designed loss function is expected to reduce the penalty on geometric factors and enhance the model’s generalization ability by minimizing interference from training interventions when there is a high degree of overlap between anchor boxes and target boxes.

To achieve this, a distance attention mechanism is introduced, and *WIoUv1* is designed with a dual-layer attention mechanism, as shown in Equation (16):

(16) $$\begin{array}{l} L_{WIoUv1}=R_{WIoU}L_{IoU}\\ R_{WIoUv1}=\exp(\frac{{\left(x-x_{gt}\right)}^{2}+{\left(y-y_{gt}\right)}^{2}}{({W_{g}}^{2}+{H_{g}}^{2})*}) \end{array}$$

where *x* and *y* represent the center coordinates of the bounding box, *x_gt_* and *y_gt_* denote the characteristics of the target box, and *W_g_* and *H_g_* refer to the dimensions of the smallest enclosing box.

To prevent the generation of gradients that impede convergence in *R_WIoUv1_*, *W_g_* and *H_g_* are separated from the calculation in the image in *WIoU*, as represented using the * operation. This effectively eliminates factors that hinder convergence, and new metrics are not introduced with this method. This approach is particularly effective when handling non-overlapping bounding boxes.

Tong et al. [55] further provide the monotonic focusing mechanism coefficient ${L^{\gamma *}}_{IoU}$ for *WIoU* for the monotonic focusing mechanism in focal loss, which can be defined using the following equation:

(17) $$L_{WIoUv2}={L^{\gamma *}}_{IoU}L_{WIoUv1}>0$$

Additionally, Equation (16) is supplemented with the coefficients of the monotonic focusing mechanism to obtain the propagation gradient of the *WIoU*, as shown in Equation (18):

(18) $$\frac{\partial L_{WIoUv2}}{\partial L_{IoU}}={L^{\gamma *}}_{IoU}\frac{\partial L_{WIoUv1}}{\partial L_{IoU}},\gamma >0$$

It is worth noting that ${L^{\gamma *}}_{IoU}$ = *r* ∈ [0, 1], where *r* represents the gradient gain. As *L_IoU_* decreases, the gradient gain *r* gradually decreases, resulting in the slower convergence of the anchor frame in the later stages of training. For the above reason, the *L_IoU_* normalization factor is introduced, as shown in Equation (19):

(19) $$L_{WIoUv2}={\left(\frac{{L^{*}}_{IoU}}{{\bar{L}}_{IoU}}\right)}^{\gamma }L_{WIoUv1}$$

where ${\bar{L}}_{IoU}$ represents the exponential running average with momentum *m*. The purpose of the introduction of the normalization factor is to retain gradient gain *r* at a high level, thus solving the problem of the slow convergence of the anchor frame in the later stages of training.

Furthermore, as all of the abovementioned *WIoU* utilize a static monotonic focusing mechanism, it is possible to induce anomalies in the anchor boxes. During training, researchers often prefer the bounding box to return to a standard quality sample anchor box. Therefore, for anchor boxes with significant outliers, a smaller gradient gain should be assigned to prevent low-quality sample anchor boxes from having a substantial impact on the gradient. Thus, the researchers developed the non-monotonic focusing coefficient *τ* and used it in Equation (16) to derive Equation (20):

(20) $$L_{WIoUv3}=rL_{WIoU},r=\frac{\tau }{\delta \alpha^{\tau -\delta }}$$

where if *τ* = *δ*, then *r* = 1. The anchor box achieves the maximum gradient gain when the anomaly of the anchor box satisfies *τ* as a set constant value. The quality classification criterion of the anchor frame changes dynamically due to the dynamic nature of *x*. This enables *WIoUv3* to adopt the most appropriate gradient gain allocation strategy for the current context at any moment, effectively optimizing the performance of the neural network model in local operation.

It is important to note that *WIoU* remains a BBR-based method. While *IoU*-based BBR methods aim to facilitate iterative convergence of the model by introducing new loss terms, they may overlook the inherent limitations of the *IoU* loss function itself. Although the *IoU* loss can effectively characterize the state of bounding box regression in theory, it does not adaptively adjust to different detectors and detection tasks, and its generalization ability is relatively limited in practice. To address these issues, Zhang et al. [56] proposed *Inner–IoU* in 2023.

*Inner–IoU* is designed to compensate for the weak convergence and slow convergence rates prevalent in loss functions widely utilized in various detection tasks. It introduces a scaling factor to control the proportional size of auxiliary bounding boxes, thus addressing the problem of weak generalization capabilities inherent in existing methods.

**Figure 11:**
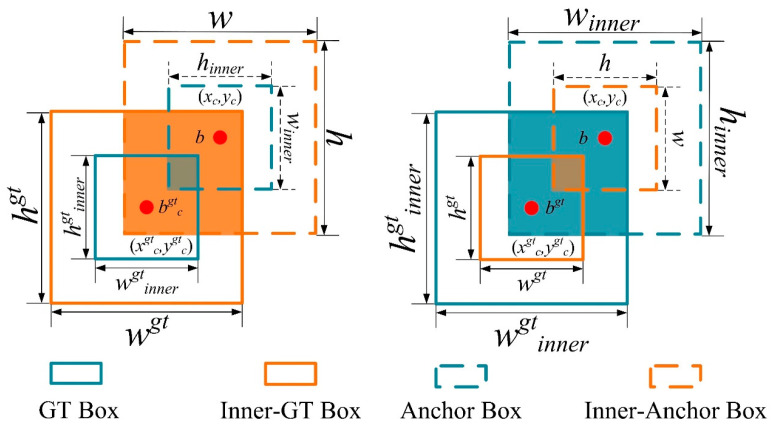
Schematic diagram of *Inner–IoU* mechanism.

The operating mechanism of *Inner–IoU* is illustrated in a schematic diagram shown in Figure 11.

In Figure 11, *b^gt^* represents the center point of the annotated data box, and *b* represents the center point of the anchor box. The coordinates of the center points for the annotated data box and the Inner annotated data box are (*x^gt^_c_,y^gt^_c_*), and the coordinates for the anchor box and Inner anchor box are (*x_c_,y_c_*). *w_gt_* and *h_gt_* represent the width and height of the annotated data box, respectively, while *w* and *h* represent the width and height of the anchor box.

The calculation formula for *Inner–IoU* is presented in Equations (21)–(27):

(21) $${b^{gt}}_{r}={x^{gt}}_{c}+\frac{w^{gt}\cdot ratio}{2},{b^{gt}}_{l}={x^{gt}}_{c}-\frac{w^{gt}\cdot ratio}{2}$$

(22) $${b^{gt}}_{b}={y^{gt}}_{c}+\frac{h^{gt}\cdot ratio}{2},{b^{gt}}_{t}={y^{gt}}_{c}-\frac{h^{gt}\cdot ratio}{2}$$

(23) $$b_{r}=x_{c}+\frac{w\cdot ratio}{2},b_{l}=x_{c}-\frac{w\cdot ratio}{2}$$

(24) $$b_{b}=y_{c}+\frac{h\cdot ratio}{2},b_{t}=y_{c}-\frac{h\cdot ratio}{2}$$

(25) $$\mathrm{inter}=(\min({b^{gt}}_{r},b_{r})-\max({b^{gt}}_{l},b_{l}))\cdot ((\min({b^{gt}}_{b},b_{b})-\max({b^{gt}}_{t},b_{t}))$$

(26) $$\mathrm{union}=(w^{gt}\cdot h^{gt})\cdot {\left(ratio\right)}^{2}+(w\cdot h)\cdot {\left(ratio\right)}^{2}-\mathrm{inter}$$

(27) $$IoU^{Inner}=\frac{\mathrm{inter}}{\mathrm{union}}$$

where the variable *ratio* represents the scaling factor, with *ratio* ∈ [0.5, 1.5]; “inter” refers to the intersection between the Inner anchor box and the Inner annotated data box; and “union” denotes the intersection of the anchor box and the annotated data box minus “inter”.

It is important to note that, although *Inner–IoU* addresses the limitations in generalization capabilities of traditional *IoU* and can accelerate the convergence speed of high-quality instance anchor boxes, the issue of slowed convergence speed for anchor boxes due to low-quality instances still arises in the later stages of training. Furthermore, it has been observed through experimentation that the strengths and weaknesses of *Inner–IoU* and *WIoU* are complementary. Consequently, *Inner–IoU* and *WIoU* are combined in this study to form *Inner–WIoU*, which integrates the advantages of both to further enhance the fitting capability of anchor boxes in neural network models.

The calculation formula for *Inner–WIoU* is presented in the following equation:

(28) $$L_{Inner-IoU}=1-IoU^{Inner}$$

(29) $$L_{Inner-WIoU}=L_{WIoUv3}+IoU-IoU^{Inner}$$

The experimental results presented in Section 4.4 prove that *Inner–WIoU*, compared to the currently latest applied *WIoU*, exhibits superior generalization capabilities and faster convergence speed.

## 4. Dataset, Experimental Results, and Discussion

In this section, a series of experiments are presented and conducted to investigate the impact of the network improvements described in Section 3 on the performance of speed bump detection tasks. The contents include the creation of datasets, the introduction of the experimental environment, evaluation metrics, and the analysis of the experimental results.

### 4.1. Dataset Production

**Figure 12:**
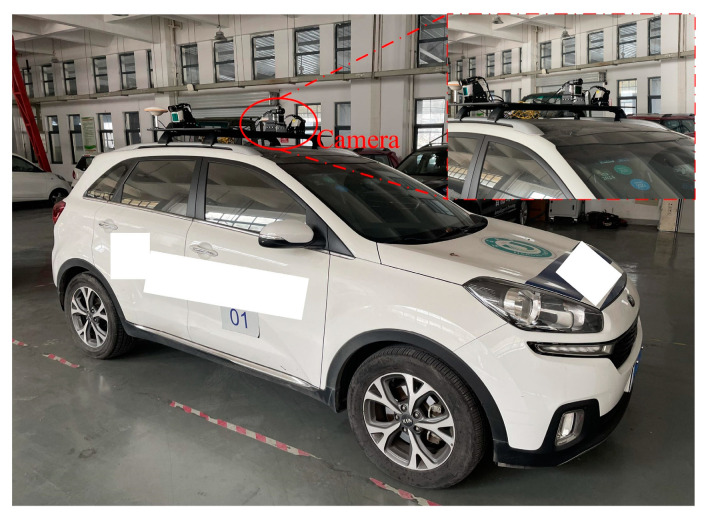
Experimental data collection vehicle.

**Figure 13:**
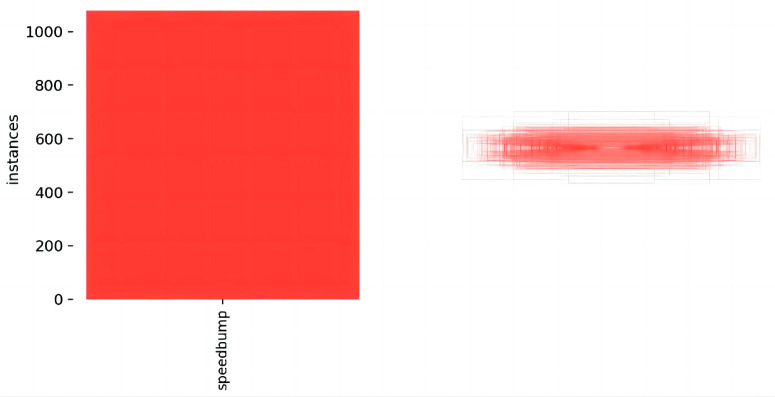
Distribution of label numbers and box shapes in the dataset.

**Figure 14:**
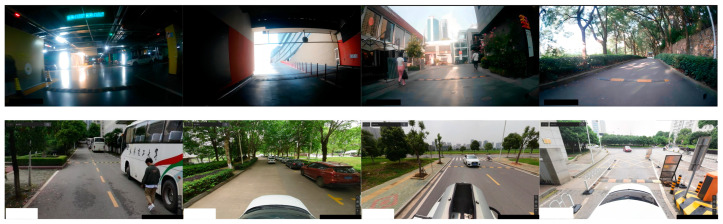
Representative images from various scenarios.

Due to the absence of a public dataset specifically for road speed bumps, a self-built dataset was created. The dataset comprises numerous photos taken in underground parking lots, public parking lots, and campus streets. The data sources include the onboard camera of the experimental data collection vehicle and Baidu Street View, with a data source ratio of 1:9. The start of the data collection period is 2012, and the end of the data collection period is 2022. Figure 12 depicts the experimental data collection vehicle. In this study, we collected 1000 images of speed bumps, which were preprocessed and stored in JPG format with a resolution of 1280 × 720. Included in these images are speed bumps with different wear conditions, including heavily worn, lightly worn, and new, to ensure diverse data. All labels were manually reviewed to ensure data accuracy. To help the model learn the features of the target more effectively, as many images as possible were assigned to the training set. The dataset was divided into training, validation, and testing sets in an 8:1:1 ratio to ensure independent subsets of data for model training and evaluation. Figure 13 displays the distribution of label numbers and box shapes in the dataset, while Figure 14 shows representative images from various scenarios.

### 4.2. Experimental Environment

The experimental platform used in this study is Ubuntu 20.04, with an Intel(R) Xeon(R) Platinum 8255C CPU @ 2.50 GHz as the central processing unit, an NVIDIA RTX 3080 (10 GB) as the graphics processing unit, CUDA version 11.3, PyTorch version 1.10.0, Python version 3.8, and 40 G of memory. The platform specifics are presented in Table 2.

The training parameter settings are consistent across models. The iteration epochs have an upper limit of 800, based on convergence observed in multiple training sessions with the self-constructed dataset. To balance GPU memory efficiency with computation speed, the batch size was set to 16. No pre-training weights were used during the training process to ensure the model was trained specifically for road speed bumps. The remaining hyperparameters were kept at their default settings.

### 4.3. Evaluation Metrics

The evaluation metrics used in this study include precision, recall, F1 score, average precision (AP), and mean average precision (mAP). When evaluating the algorithms in this study, speed bumps are considered as positive instances, and non-speed bumps as negative instances. The formulas for each of these evaluation metrics are presented in Equations (30)–(34).

(30) $$\mathrm{Precision}=\frac{TP}{TP+FP}$$

(31) $$\mathrm{Recall}=\frac{TP}{TP+FN}$$

(32) $$F1=\frac{2\times \mathrm{Precision}\times \mathrm{Recall}}{\mathrm{Precision}+\mathrm{Recall}}$$

(33) $$\mathrm{AP}=\int_{0}^{1}\mathrm{Precision}\mathrm{smooth}\left(\mathrm{Recall}\right)d\left(\mathrm{Recall}\right)$$

(34) $$\mathrm{mAP}=\frac{1}{N}\sum_{i=1}^{N}{\mathrm{AP}}_{i}$$

where True Positive (*TP*) is the number of accurately detected annotated speed bump bounding boxes, False Positive (*FP*) is the number of bounding boxes incorrectly marked as speed bumps, and False Negative (*FN*) is the number of speed bump bounding boxes that were not detected.

Precision represents the proportion of accurately located speed bumps among all detection results, while recall indicates the proportion of accurately located speed bumps among all annotated speed bump bounding boxes in the test set. Precision reflects the false positive rate, i.e., the proportion of samples that are actually negative among all those judged as positive by the model. Recall, on the other hand, reflects the miss rate, i.e., the proportion of samples correctly identified as positive among all actual positive samples. AP represents the precision–recall curve, with the area under the curve used to measure the average precision of the test results. mAP is the average of AP, used to comprehensively evaluate the overall performance of the algorithm across the entire dataset. These evaluation metrics are commonly used performance indicators in target detection algorithms.

Since both mAP and mAP50_95 evaluate the precision and recall of the model and assess its performance at different IoU thresholds, they provide a comprehensive reflection of the model’s performance [57,58,59]. Additionally, evaluating the model under a range of higher IoU thresholds ensures that it not only recognizes objects well but also has high localization accuracy. Therefore, this paper uses mAP and mAP50_95 as evaluation metrics in the following comparative experiment and ablation study.

### 4.4. Experimental Results and Discussion

#### 4.4.1. Comparative Experimental Results of Self-Built Datasets

Compared to YOLOv3-tiny and YOLOv7-tiny, YOLOv5s-FPNet demonstrates significant advantages in terms of performance metrics. Specifically, YOLOv5s-FPNet improves mAP by 9.51% and 73.14%, respectively. Additionally, its performance in mAP50_95 is impressive, with improvements of 28.42% and 92.76%, respectively. The results are given in Table 3.

**Figure 15:**
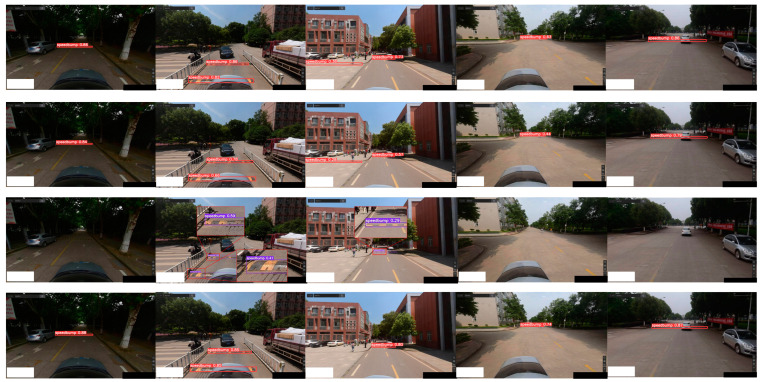
YOLOv5s, YOLOv3-tiny, YOLOv7-tiny, and YOLOv5-FPNet speed bump detection results under normal lighting conditions. The first row illustrates the speed bump detection results of YOLOv5s. The second row illustrates the speed bump detection results of YOLOv3-tiny, the third row illustrates the speed bump detection results of YOLOv7-tiny, and the fourth row illustrates the speed bump detection results of YOLOv5-FPNet.

**Figure 16:**
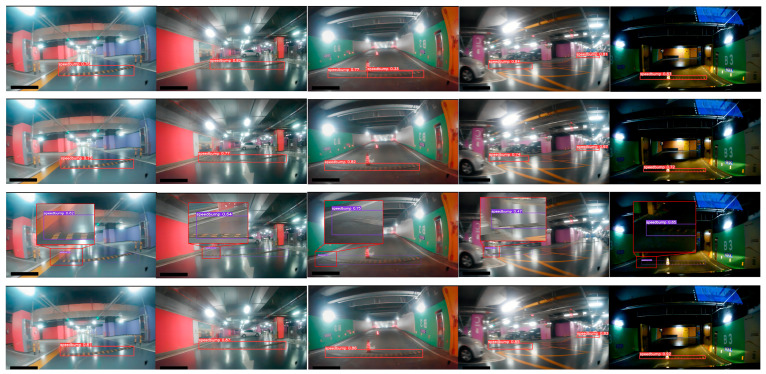
YOLOv5s, YOLOv3-tiny, YOLOv7-tiny, and YOLOv5-FPNet speed bump detection results under dark lighting conditions. The first row illustrates the speed bump detection results of YOLOv5s. The second row illustrates the speed bump detection results of YOLOv3-tiny, the third row illustrates the speed bump detection results of YOLOv7-tiny, and the fourth row illustrates the speed bump detection results of YOLOv5-FPNet.

The visualization of the test results under normal lighting conditions is shown in Figure 15, and the results under dark lighting conditions are shown in Figure 16.

The experiments mentioned above demonstrate that YOLOv5 and YOLOv5-FPNet, proposed in this paper, perform better than YOLOv3-tiny and YOLOv7-tiny.

**Figure 17:**
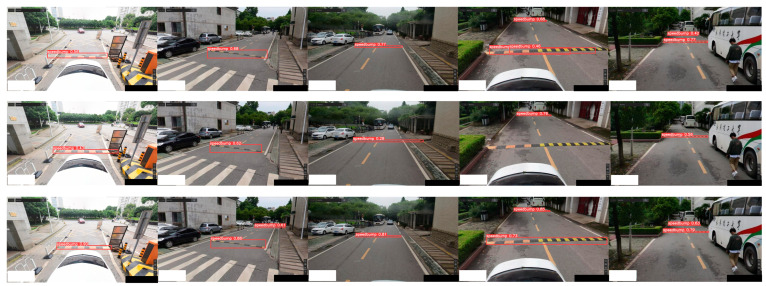
YOLOv5, YOLOv5–FasterNet, and YOLOv5-FPNet speed bump detection results under normal lighting conditions. The first row illustrates the speed bump detection results of YOLOv5. The second row illustrates the speed bump detection results of YOLOv5–FasterNet, and the third row illustrates the speed bump detection results of YOLOv5-FPNet.

As shown in Figure 17, speed bumps can be detected under normal lighting conditions. The performance evaluation metrics include mAP, mAP50_95, and frames per second (FPS). Table 4 presents the comparative experimental results of the proposed YOLOv5-FPNet against current mainstream lightweight networks.

Based on the data given in Table 4, the proposed YOLOv5-FPNet demonstrates significant superiority in terms of performance metrics compared to lightweight object detection models that utilize FasterNet, MobileNet, GhostNet, EfficientNet, and ShuffleNet as their backbone networks. Specifically, YOLOv5-FPNet achieved improvements in mAP of 12.85%, 38.76%, 23.66%, 13.90%, and 12.55%, respectively. Furthermore, in terms of mAP50_95, its performance was equally impressive, showing increases of 15.98%, 143.15%, 40.06%, 39.24%, and 27.05%. In addition, in terms of FPS, the enhancements reached −7.07%, 51.23%, 44.13%, 50.61%, and 17.20%, respectively.

Drawing from the data presented, it can be concluded that YOLOv5-FPNet demonstrates a significant enhancement in performance on speed bump detection tasks compared to the existing lightweight networks, and it has also made substantial advancements in the real-time aspects of speed bump detection tasks.

As illustrated in Figure 17, this study compares the visualization results of speed bump detection using YOLOv5, YOLOv5–FasterNet, and YOLOv5-FPNet.

From Figure 17, it can be observed that under normal lighting conditions, higher confidence levels, greater accuracy in the convergence of bounding boxes, and significant reductions in both false positive rates and miss rates are exhibited with the proposed network. Table 5 shows a comparison between confidence and detection accuracy in the five examples.

**Figure 18:**
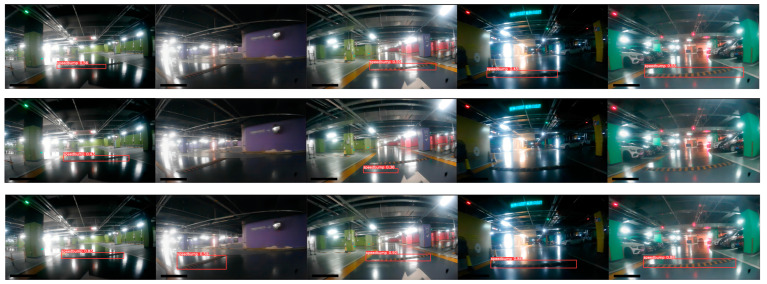
YOLOv5, YOLOv5–FasterNet, and YOLOv5-FPNet speed bump detection results under dark lighting conditions. The first row illustrates the speed bump detection results of YOLOv5. The second row illustrates the speed bump detection results of YOLOv5–FasterNet, and the third row illustrates the speed bump detection results of YOLOv5-FPNet.

Furthermore, the visualization effects of speed bump detection under poor lighting conditions, such as in underground parking lots, are compared among the aforementioned three networks, as depicted in Figure 18.

It is evident from the comparison that the proposed network demonstrates superior detection performance under low-light conditions, in contrast to both YOLOv5 and YOLOv5–FasterNet. Table 6 shows a comparison between the confidence and detection accuracy in the five examples.

**Figure 19:**
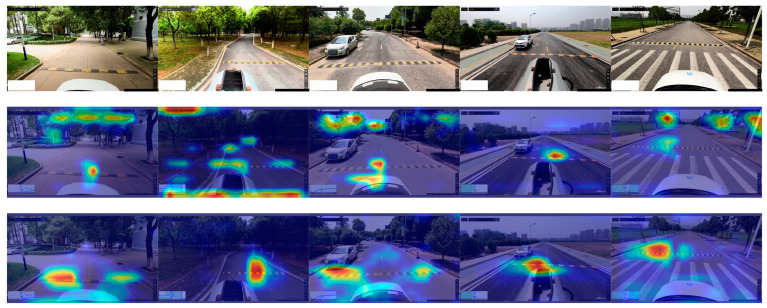
Heatmap comparison of YOLOv5-FPNet with and without SimAM. The first row illustrates the original plot of the dataset. The second row illustrates YOLOv5-FPNet without SimAM. The third row illustrates YOLOv5-FPNet with SimAM. The darker the color, the higher the impact on the confidence of the result.

This study delved deeper by comparing the heatmap outputs of YOLOv5-FPNet integrated with and without the SimAM attention mechanism. This comparison was conducted using Gradient-weighted Class Activation Mapping (GradCAM). The visualization of the experimental results is presented in Figure 19.

As can be discerned from Figure 19, in comparison to YOLOv5-FPNet devoid of the SimAM attention mechanism, the integration of SimAM facilitates more efficacious concentration and extraction of speed bump features with the neural network model. The introduction of the SimAM attention mechanism substantially augments the proficiency of YOLOv5-FPNet in delineating features pertinent to speed bumps, effectively mitigating the perturbations induced by extraneous road elements.

To further elucidate the impact of the ratio parameter in *Inner–WIoU*, this research undertook a comprehensive series of experimental analyses, the outcomes of which are methodically delineated in Table 7.

An analysis of Table 7 elucidates that *Inner–WIoU* exerts a beneficial influence on the convergence and generalization capabilities of anchor boxes vis-à-vis *WIoU*. Furthermore, it is discerned that modulation of the ratio parameter significantly impacts the model’s proficiency in terms of convergence and generalization. In the end, a ratio of 1.4 was chosen for this paper because it has been found to provide optimal convergence.

Drawing from the aforementioned comparative analyses, it can be inferred that the proposed YOLOv5-FPNet manifests exemplary efficacy in the domain of speed bump detection, showcasing enhanced performance relative to the prevailing lightweight network architectures in the field.

#### 4.4.2. Ablation Study

To quantitatively ascertain the contributory impact of individual components within the described neural network architecture, a meticulously structured series of ablation study analyses were undertaken. The objective of these analyses was to rigorously substantiate the efficacious role played by the newly integrated enhancement modules in amplifying the detection accuracy of speed bumps. The empirical findings derived from these ablation experimental analyses are systematically cataloged in Table 8.

In the ablation study, the baseline model selected was the original YOLOv5s. As indicated in Table 3, the introduction of optimization modules resulted in varying degrees of performance enhancement in the neural network model for speed bump detection tasks. By transitioning the backbone to FPNet, there was a notable reduction in the model’s computational load, with a 28.93% decrease in floating-point operation volume and a 20.43% reduction in the parameter count. However, this architectural adjustment led to a decrease in model precision, as evidenced by a 2.86% reduction in mAP and an 8.44% decrease in mAP50_95.

The C3-SFC module efficaciously augmented precision within the context of speed bump detection tasks. This enhancement was achieved with a marginal increment in the number of parameters of 3.09% while the floating-point computational load remained unchanged. Concurrently, this architectural innovation contributed to a commendable enhancement in model performance metrics, as evidenced by 4.09% and 2.84% increases in mAP and mAP50_95, respectively.

The incorporation of the SimAM attention mechanism between the backbone and SPPF, along with SimAM integration into the large object detection head, substantially bolstered the neural network model’s capacity for learning and discerning features within the ambit of speed bump detection endeavors. With an increment of 0.88% in floating-point operation and 0.59% in parameters, there was an enhancement of 1.35% in mAP and a 3.91% improvement in mAP50_95.

The optimization of the loss function, by substituting the default IoU with Inner–WIoU, facilitated respective enhancements of 3.27% and 5.97% in mAP and mAP50_95, significantly augmenting the convergence and generalization capabilities of the bounding boxes.

Aggregating the outcomes of the aforementioned ablation experiments, the refined YOLOv5-FPNet, in comparison to the baseline model, resulted in reductions of 17.77% in parameter count and 28.30% in floating-point operation volume, alongside increases of 5.83% and 3.68% in mAP and mAP50_95, respectively.

The conducted ablation experiments demonstrate that the proposed combination can assist autonomous vehicles in achieving faster and more precise speed bump detection, thereby providing passengers and drivers with a safer and more comfortable autonomous driving experience.

## 5. Conclusions

In this study, an enhanced algorithm based on the YOLOv5 model, termed YOLOv5-FPNet, is introduced to tackle the multifaceted challenges encountered by the current YOLOv5 object detection algorithm in speed bump detection. These challenges encompass issues such as image aberration, feature loss, and varying lighting conditions. To address the inadequacy in feature extraction capabilities during image aberration, the algorithm integrates the Dynamic Snake Convolution operator (DSConv) into FasterNet. Furthermore, through the refinement of the C3 module and the loss function, coupled with the integration of a parameter-free attention mechanism, SimAM, an augmentation in the model’s ability to extract features under the conditions of feature erosion and suboptimal illumination is achieved. These enhancements substantially contribute to the fortification of the model’s robustness. The empirical outcomes elucidate that, in comparison to the lightweight neural network backbones prevalently employed in contemporary applications, the proposed YOLOv5-FPNet confers superior precision and enhanced real-time performance in speed bump detection tasks. Relative to the foundational YOLOv5 architecture, the proposed YOLOv5-FPNet achieves a better balance between accuracy and real-time performance. Furthermore, this model demonstrates the capacity for extended applicability across a broader spectrum of road-based object detection tasks.

Despite the efficiency demonstrated with the proposed YOLOv5-FPNet model in the context of speed bump detection, certain constraints persist. Specifically, in terms of discerning distantly located speed bumps (akin to small object detection), despite a notable enhancement over the baseline YOLOv5 object detection paradigm, the performance remains suboptimal. To further advance the dependability of speed bump detection methodologies, with the aim of elevating safety and comfort levels in autonomous vehicle drive experience, future research should focus on the following areas:

i. Dataset Enhancement: Pursuing the systematic aggregation of an extensive and varied dataset, with a focus on augmenting the representation of small targets, thereby facilitating increases in the precision of the target detection algorithm.
ii. Multimodal Perception Fusion: Employing a multimodal perception framework, encompassing the synthesis of sensory data from optical imaging devices, Light Detection and Ranging (LiDAR) apparatus, and millimeter-wave radar systems to enhance the detection robustness of small-scale targets across diverse environments.
iii. Algorithm Enhancement: Pruning and distillation algorithms could be adopted to further improve the real-time performance and accuracy of the model.

## Figures and Tables

**Table 1:** Results of convolutional kernel performance comparison experiment.

	Parameters	FLOPs	All Time	Mean Time	FPS
Conv2D	147.840 kB	77.578 G	23.34170	0.00778	128.52534
DWConv2D [32]	18.304 kB	9.731 G	23.03476	0.00768	130.23795
GhostConv2D [38]	9.152 kB	4.865 G	18.68083	0.00623	160.59240
GSConv2D [48]	75.712 kB	39.762 G	28.60418	0.00953	104.87976
DCNV2 [46]	31.387 kB	16.576 G	124.84611	0.04162	24.02958
DCNV3 [49]	38.171 kB	20.133 G	72.85585	0.02429	41.17720
DSConv [47]	55.250 kB	25.765 G	22.47875	0.05683	126.49330

**Table 2:** Test environment.

Name	Version
OS	Ubuntu20.04
CPU	Intel(R) Xeon(R) Platinum 8255C CPU @ 2.50 GHz
GPU	NVIDIA RTX 3080 (10 GB)
CUDA	11.3
PyTorch	1.10.0
Python	3.8
RAM	40 G

**Table 3:** YOLOv3-tiny, YOLOv7-tiny, YOLOv5s and YOLOv5s-FPNet comparison experimental results. ↑ denotes the improvement of the comparison algorithm, ↓ denotes the degradation of the comparison algorithm.

Model	mAP/%	mAP50_95/%	FPS
YOLOv3-tiny	77.8(↓9.51%)	37.3(↓28.42%)	113.1(↑2.39%)
YOLOv7-tiny	49.2(↓73.14%)	24.9(↓92.76%)	112.3(↑1.69%)
YOLOv5s	80.5(↓5.52%)	46.2(↓2.19%)	112.5(↑2.70%)
YOLOv5s-FPNet (ours)	85.2	47.9	110.4

**Table 4:** Lightweight network comparison experimental results. ↑denotes the enhancement of the proposed algorithm, ↓ denotes the degradation of the proposed algorithm.

Model	mAP/%	mAP50_95/%	FPS
YOLOv5s–FasterNet	75.5 (↑12.85%)	41.3 (↑15.98%)	118.8 (↓7.07%)
YOLOv5s–MobileNet	61.4 (↑38.76%)	19.7 (↑143.15%)	73.0 (↑51.23%)
YOLOv5s–GhostNet	68.9 (↑23.66%)	34.2 (↑40.06%)	76.6 (↑44.13%)
YOLOv5s–EfficientNet	74.8 (↑13.90%)	34.4 (↑39.24%)	73.3 (↑50.61%)
YOLOv5s–ShuffleNet	75.7 (↑12.55%)	37.7 (↑27.05%)	94.2 (↑17.20%)
YOLOv5s-FPNet (ours)	85.2	47.9	110.4

**Table 5:** Comparison of sample image detection results under normal illumination.

Figure	Algorithm	Confidence	Miss Detection
1	YOLOv5s	0.56	0
1	YOLOv5s–FasterNet	0.43	0
1	YOLOv5s-FPNet	0.9	0
2	YOLOv5s	0.68/0	1
2	YOLOv5s–FasterNet	0.62/0	1
2	YOLOv5s-FPNet	0.88/0.61	0
3	YOLOv5s	0.77	0
3	YOLOv5s–FasterNet	0.28	0
3	YOLOv5s-FPNet	0.81	0
4	YOLOv5s	0.46/0.68	0
4	YOLOv5s–FasterNet	0/0.76	1
4	YOLOv5s-FPNet	0.73/0.85	0
5	YOLOv5s	0.77/0.42	0
5	YOLOv5s–FasterNet	0.56/0	1
5	YOLOv5s-FPNet	0.79/	0

**Table 6:** Comparison of sample image detection results under dark illumination.

Figure	Algorithm	Confidence	Miss Detection
1	YOLOv5s	0.56	0
1	YOLOv5s-FasterNet	0.43	0
1	YOLOv5s-FPNet	0.83	0
2	YOLOv5s	0	1
2	YOLOv5s-FasterNet	0	1
2	YOLOv5s-FPNet	0.59	0
3	YOLOv5s	0.59	0
3	YOLOv5s-FasterNet	0	1
3	YOLOv5s-FPNet	0.92	0
4	YOLOv5s	0.45	0
4	YOLOv5s-FasterNet	0	1
4	YOLOv5s-FPNet	0.86	0
5	YOLOv5s	0.55	0
5	YOLOv5s-FasterNet	0	1
5	YOLOv5s-FPNet	0.86	0

**Table 7:** Experimental effect of ratio on model performance.

Loss Function	mAP/%	mAP50_95/%
WIoU	84.3	45.9
Inner–WIoU (1.3)	84.7 (+0.3)	46.3 (+0.4)
Inner–WIoU (1.4)	85.2 (+0.9)	46.7 (+0.8)
Inner–WIoU (1.5)	84.8 (+0.4)	46.7 (+0.8)

**Table 8:** Ablation experimental results. √ represents that the module is used. × represents that the module is not used.

Baseline	FPNet	C3-SFC	SimAM	Inner–WIoU	FLOPS	Params/M	mAP/%	mAP50_95/%
√	×	×	×	×	15.9 G	6.69	80.5	46.2
√	√	×	×	×	11.3 G	5.33	78.2	42.3
√	√	√	×	×	11.3 G	5.49	81.4	43.5
√	√	√	√	×	11.4 G	5.53	82.5	45.2
√	√	√	√	√	11.4 G	5.53	85.2	47.9

## Data Availability

The data presented in this study are available on request from the corresponding author.
